# To predict the radiosensitivity of nasopharyngeal carcinoma using intravoxel incoherent motion MRI at 3.0 T

**DOI:** 10.18632/oncotarget.17367

**Published:** 2017-04-21

**Authors:** Wen Bo Chen, Bin Zhang, Long Liang, Yu Hao Dong, Guan Hui Cai, Chang Hong Liang, Bo Wen Lan, Shui Xing Zhang

**Affiliations:** ^1^ Department of Radiology, Guangdong Academy of Medical Sciences/Guangdong General Hospital, Guangzhou, Guangdong, P.R. China; ^2^ Department of Radiology, HuiZhou Municipal Central Hospital, Huizhou, Guangdong, P.R. China; ^3^ Southern Medical University, Guangzhou, Guangdong, P.R. China; ^4^ Shantou University Medical College, Shantou, Guangdong, P.R. China

**Keywords:** intravoxel incoherent motion, MRI, nasopharyngeal carcinoma, IMRT, radiosensitivity

## Abstract

**Purpose:**

To investigate intravoxel incoherent motion (IVIM) MRI for evaluating the sensitivity of radiotherapy on nasopharyngeal carcinoma (NPC).

**Results:**

The reproducibility between intra-observer and inter-observer was relatively good. *D* (0.72×10^−3^ mm^2^/s±0.14 *vs*. 0.54×10^−3^ mm^2^/s±0.23; *P* < 0.001) and D* (157.92×10^−3^ mm^2^/s±15.21 *vs*. 120.36×10^−3^ mm^2^/s±10.22; *P* < 0.0001) were significantly higher in effective group than poor-effective group, whereas the difference of *f* (18.79%±2.51 *vs*. 16.47%±1.51) and *ADC* (1.21×10^−3^ mm^2^/s±0.11 *vs*. 1.33×10^−3^ mm^2^/s±0.23) could not reach statistical significant between the 2 groups (*P* > 0.05).

**Conclusions:**

IVIM may be potentially useful in assessing the radiosensitivity of NPC. The higher *D* value combining with higher D* value might indicate the more radiosensitive of NPC, and increased D* might reflect increased blood vessel generation and parenchymal perfusion in NPC.

**Materials and Methods:**

Sixty consecutive patients (20 female, range, 27-83 years, mean age, 52 years) newly diagnosed NPC in the stage of T3 or T4 were enrolled. Forty-two of them were divided into effective group clinically after a standard radiotherapy according to the RECIST criteria. IVIM with 13 b-values (range, 0-800 s/mm^2^) and general MRI were performed at 3.0T MR scanner before and after radiotherapy. The parameters of IVIM including perfusion fraction (*f*), perfusion-related diffusion (D*), pure molecular diffusion (*D*) and apparent diffusion coefficient (*ADC*) were calculated. Two radiologists major in MRI diagnose analyzed all images independently and placed regions of interest (ROIs). Intra-class correlation coefficient (ICC) was used to evaluate intra-observer and inter-observer agreement. And Mann–Whitney test was used to assess the differences between the two groups.

## INTRODUCTION

Nasopharyngeal carcinoma (NPC) is a geographic and racial malignancy in global nation, especially in southern China with a high incidence up to 15–24/100,000 [1]. Nowadays, radiotherapy plays a very important role in the treatment of NPC, and intensity-modulated radiotherapy (IMRT) is regarded as a major breakthrough. However, a long-term outcome of large NPC series suggested up to 20% patients failed because of distant metastasis (DM) after IMRT [2]. For the failed patients, a taxane-based chemotherapy would be used to reduce DM. Therefore, it is obvious that a non-invasive and accurate technique for evaluating the radiosensitivity of NPC is urgent and can help determine whether chemotherapy or immunotherapy combination is needed or not.

Due to its superior soft tissue contrast resolution, general MRI was mainly used for morphological diagnosis and staging of NPC. Unfortunately, with the defect in assessing the tumor microenvironment, both general MRI and CT are limited in evaluating the radiosensitivity of NPC. The microenvironment of tumor including interstitial hypertension and hypoxia is associated with DM and treatment failure [3, 4]. While emerging clinical data suggests that alleviating tumor hypoxia by improving tumor perfusion or oxygenation may actually enhance the outcome of radiotherapy, chemotherapy, and immunotherapy [5]. And oxygenation depends on both O2 diffusion and blood perfusion. Therefore, the tumor microenvironment including diffusion and perfusion can probably reveal the radiosensitivity of NPC.

Diffusion-weighted (DW) MR imaging enhances contrast ratio in the tissues based on the different water diffusion in distinct tissues. Besides, DWI could be used to differentiate the histological types in certain malignancies [6–8]. However, the effect of diffusion in tissues can substantially be confounded by perfusion because of the Brownian motion of H+ in pseudorandom capillary networks [9]. IVIM, a special DWI technique with several b values, can separate quantificational effects of diffusion and perfusion [10, 11]. Many studies revealed that IVIM was advantageous in demonstrating superior results in head and neck cancers compared with general DWI [12–14]. Therefore, IVIM was much more advantageous and useful for evaluating the radiosensitivity of NPC than general DWI.

According to IVIM theory [9], MR signal attenuation could be expressed using four parameters (f, D, D*, ADC) in a biexponential equation [15]. Pilot studies explored the value of IVIM in head and neck cancers [14, 16]. And in our prior study [17], we had found *D* (*P*=0.001) and *f* (*P*<0.0001) were significantly lower in patients with NPC than enlarged adenoids, whereas D* was significantly higher (*P*<0.0001), and increased D* indicated blood vessel generation and increased parenchymal perfusion in primary NPC. Although we had established a system using IVIM DWI to differentiate the tumors in nasopharyngeal region, while a very few published studies had explored IVIM in evaluating the radiosensitivity of NPC. Thus, the aim of this research is to obtain the D, D*, ADC and *f* values using IVIM theory and explore the value of IVIM in evaluating the radiosensitivity of NPC.

## RESULTS

**Figure 1:**
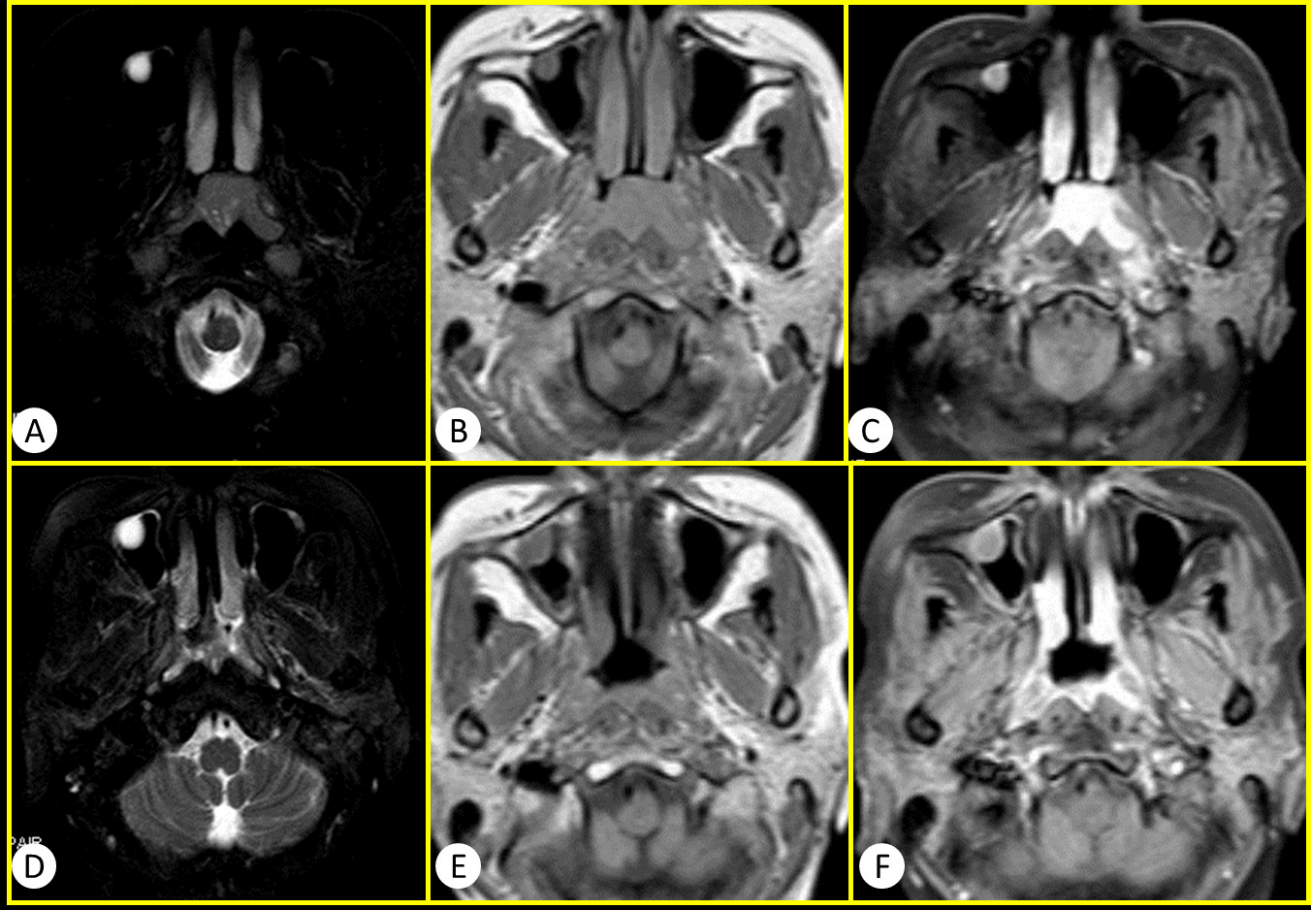
The conventional MR images of a 50 years old woman diagnosed as NPC **A.-C.** were before the therapy of IMRT and **D.-F.** were after the therapy of IMRT. A and D were T_2_WI SPAIR. B and D were T_1_WI. C and F were T_1_WI enhanced. Compared with the images before IMRT, the longest diameter of tumor lesion was obviously diminish >50% after the therapy of IMRT. Therefore this case was sort into the effective group with PR according to the RECIST standard.

**Figure 2:**
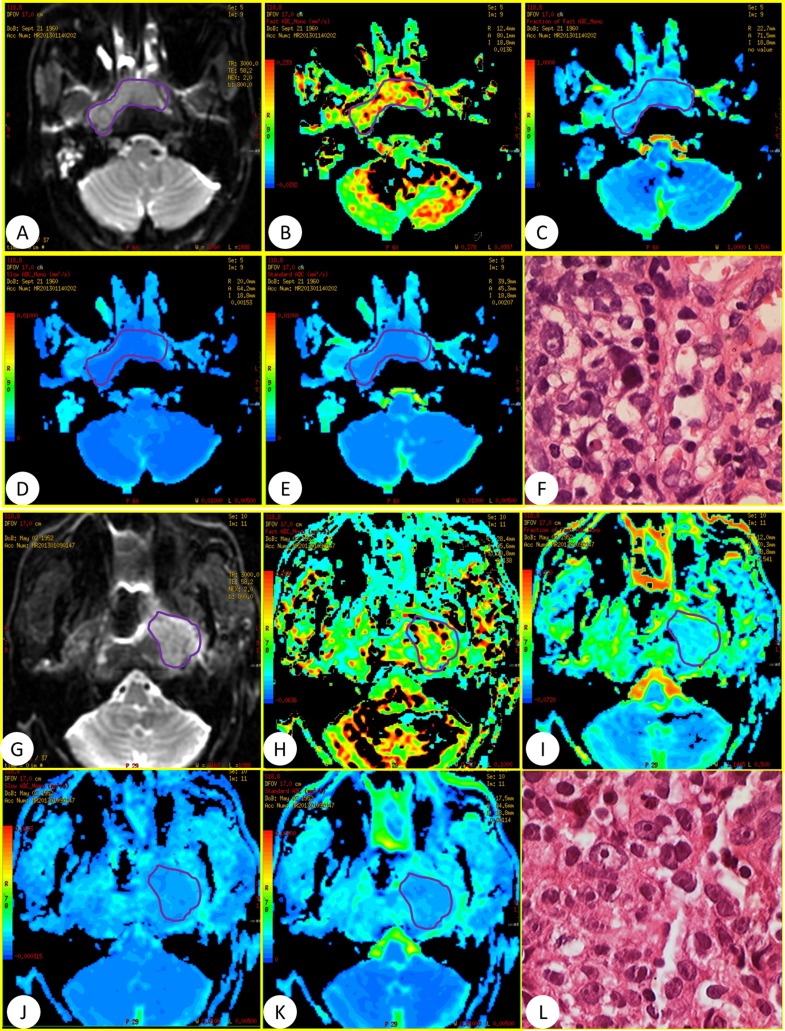
(**A–F**) Was a 52-year-old man in effective group and (**G–L**) was a 60-year-old women in poor effective group who referred to our department for confirmation of the NPC diagnosis. IVIM with 13 b values (in the range 0–800 s/mm2) was performed before standard regimen of IMRT. (A and G) DWI image, (B and H) D*, (C and I). f, (D and J) D, (E and K): ADC parameters, (F and L) pathological slides.

**Figure 3:**
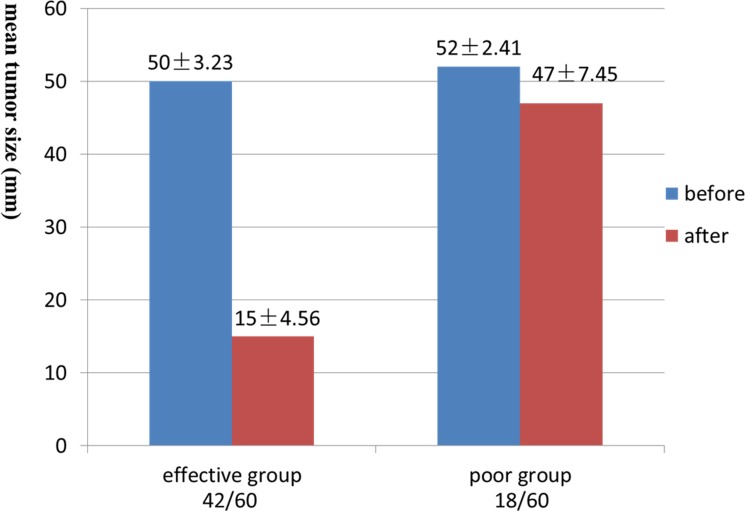
The mean tumor size before and after IMRT The mean largest diameter of lesions in effective group after IMRT was significantly decrease compared with before (50±3.23 mm vs. 15±4.56 mm, *P* < 0.05). And the difference in the poor group could not reach statistical significance (52±2.41 mm vs. 47±7.45 mm, *P* > 0.05).

General MRI and IVIM were successfully performed in the 60 patients using 3.0-T whole-body system (Signa EXCITE HD, GE Healthcare, Milwaukee, WI, USA) before and after standard regimen of IMRT. The images of general MR sequence were showed in Figure 1. IVIM images and representative pathological slides of biopsy for both effective groups and poor effective groups were showed in Figure 2. The mean largest diameters of tumor (± SD) in patients with NPC before and after IMRT were showed in Figure 3, respectively.

There were excellent inter-observer agreements in IVIM parameters *D* with ICC value of 0.937 (95% CI: 0.926–0.968) and *f* with ICC value of 0.922 (95% CI: 0.920–0.962), and a relatively good ICC value of D* with 0.902(95% CI: 0.851–0.909). The result suggested excellent agreement between the 2 readers for all IVIM measures.

The values of the IVIM parameters for NPC of the two groups before IMRT were showed in Table 1. The classification results obtained from Leave-one out tests of original grouped cases and cross-validated grouped cases were 95.0% and 93.3%.

**Table 1:** The values of the IVIM parameters for NPC of the two groups before IMRT

	D (×10^−3^mm^2^/s)	D*(×10^−3^mm^2^/s)	f(%)	ADC(×10^−3^mm^2^/s)
Effective group	0.72±0.14	157.92±15.21	18.79±2.51	1.21±0.11
(42/60)				
Poor group	0.54±0.23	120.36±10.22	16.47±1.51	1.33±0.23
(18/60)				
P value	<0.001^※^	<0.0001^※^	>0.05 >0.05

Note: Unless otherwise indicated, data are means±standard deviations.

* Mann–Whitney test for differences in IVIM parameters between the two groups.

**Figure 4:**
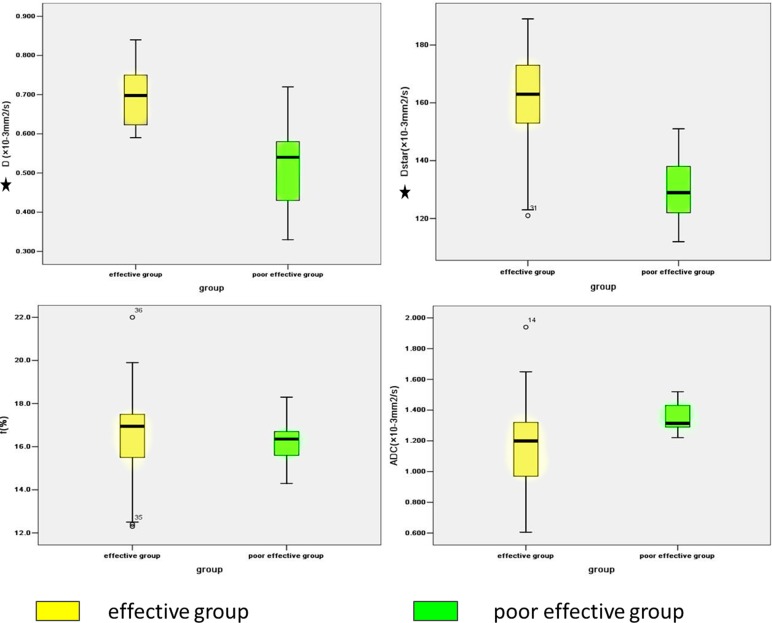
Box plots comparing *D*, D*, *f* and A*DC* between the two groups From the box plots, it is obvious that *D* and D* were significantly higher in effective group than poor-effective group, whereas the difference of *f* and *ADC* could not reach statistical significant. *means *P* < 0.05.

**Figure 5:**
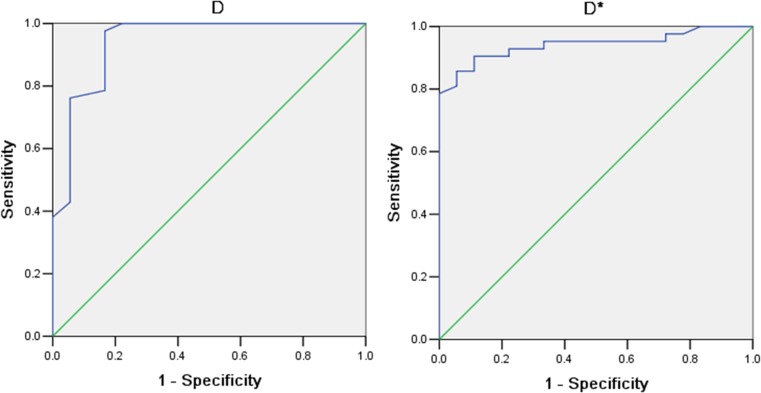
The ROC cure of *D* and D* of the two groups Our data shows when both sensitivity and specificity were adjusted to produce the highest accuracy, the optimal *D* and D* threshold for distinguishing radiosensitive NPC were 0.593×10^−3^ mm^2^/s and 147.5×10-3 mm^2^/s respectively. Besides the AUC for D* (0.942) was a little larger than *D* (0.941).

**Figure 6:**
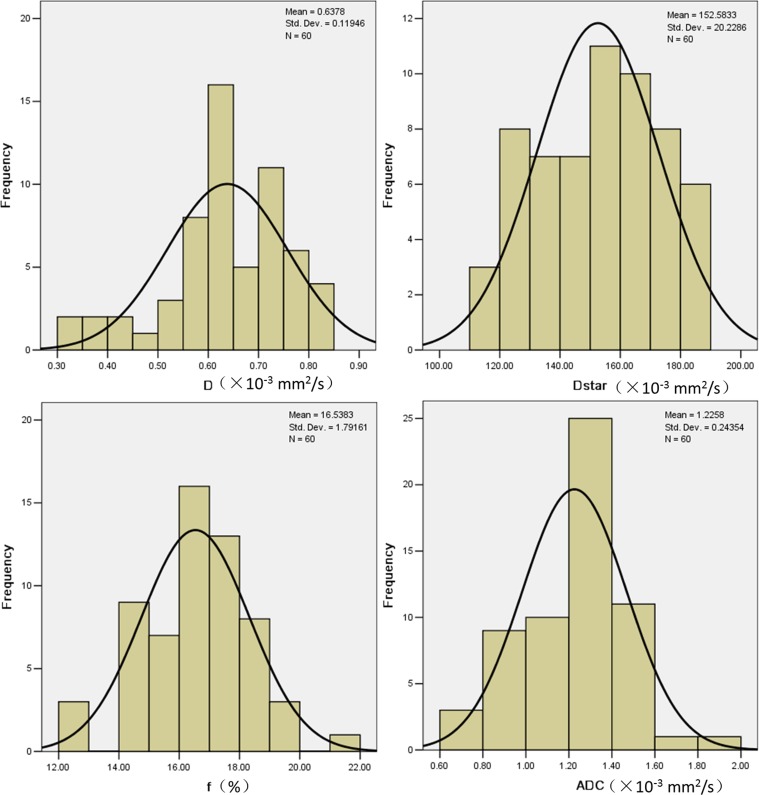
Histogram analysis was performed to to display the values of all the parameters and their distribution From this histogram, it is much in evidence that all the values of D, D* and *f* were relatively stable and acceptable, except *ADC* values. The *ADC* values of 5/18 patients in poor effective group were quirky high which made the mean ADC value illogic high.

Box plots comparing D, D*, f and *ADC* between the two groups are shown in Figure 4. As shown in Figure 5, the ROC curves indicated that when both sensitivity and specificity were adjusted to produce the highest Youden index, the optimal *D* and D* threshold for distinguishing radiosensitive NPC were 0.593×10^−3^ mm^2^/s and 147.5×10^−3^ mm^2^/s respectively. Besides the AUC for D* (0.942) was a little larger than *D* (0.941). The sensitivity of *D* and D* were 93.33% and 97.37%. The specificity of *D* and D* were 60% and 50%. Histogram analysis (Figure 6) demonstrated that all the values of *D*, D* and *f* were relatively stable and acceptable, while *ADC* values of 5/18 in poor effective group were quirky and illogic high.

## DISCUSSION

Comparing to the general DWI model, the IVIM fits the signal decay with a biexponential decay and the parameters may reflect water diffusion and blood perfusion more accurately [9]. By this model, the parameters (D, f, D*, *ADC*) are estimated simultaneously; *f* and D* are related to blood perfusion, and *D* and *ADC* are related to water diffusion [18–20]. The IVIM model was recently applied to investigate human placental function, renal perfusion, characterize prostate and breast tumors [21–24]. However, we applied the IVIM model to evaluate the radiosensitivity of NPC. In our study, local shim, parallel imaging, and a reduced echo time were used to insure high quality IVIM imaging, which was confirmed to be useful in our prior research [17].

Using an 11-b DW imaging sequence, Guiu et al. [25] reported that seven b values (0, 5, 15, 25, 35, 50 and 100 s/mm^2^) could model the first portion of the bi-exponential decay curve. The number of b-values used for extracting perfusion-sensitive information during DWI varies between studies and ranges from four to more than ten. A larger number of b-values provided more powerful data supporting the estimate and, in particular, enabling the uncertain parameter to be measurable. The accuracy of estimating D* improved with sampling (i.e., as the number of b-values increased) in the 0–200 s/mm^2^ range [26, 27]. Susceptibility artifacts increase with the use of higher b-values, typically around 1000 s/mm^2^. Furthermore, the poor signal-to noise ratio (SNR) obtained at higher b-values decreases the accuracy of IVIM calculations. Consequently, we tried to minimize this effect by setting the maximum b-value to be 800s/mm^2^. In our study, 13-b values were used, and nine of these were applied to model this region of the bi-exponential curve. As a result, the reproducibility of the IVIM parameters both in intra-observer and inter-observer was relatively good.

In our study, we found that *D* value was significantly higher in effective group than in poor-effect group (0.72×10^−3^ mm^2^/s±0.14 vs. 0.54×10^−3^ mm^2^/s±0.23; *P*<0.001), indicating that *D* was restricted in the NPC cases with poor radiosensitivity. Our finding was similar with other studies indicated cancers present a greater impediment to diffusion due to the more densely packed tumor cells and more cell membranes. In other words, the decrease in the number of cells or necrosis during treatment leaded to smaller impediment to diffusion [28, 29]. Simultaneously, the data suggested that IVIM had greater clinical potential to assess the radiosensitivity of tumors based on their *D* values with a threshold of 0.593×10^−3^ mm^2^/s. The significant differences of *D* values between the two groups of NPC may due to the differences of cellularity and extracellular matrix composition. In the present preclinical study [30], tumors subjected to DW-MRI were examined with respect to cell density, fraction of hypoxic tissue, level of interstitial hypertension, and metastatic status. The low *D* values were found to have high cell densities, indicating restricted Brownian motion of water [31–33]. The increased cellularity and varying amounts of stromal tissues would reduce molecular diffusivity [34], the same as oxygen diffusion and cause hypoxia which resulted in the poor radiosensitivity of IMRT in NPC. Therefore, *D* might be useful in clinical practice to help evaluate the radiosensitivity of NPC before IMRT. On the other hand, despite the statistically significant differences in the overall *D* between the two groups, the D values vary considerably and, importantly, overlapped appreciably between the two groups of cases.

Normally, *D* and *ADC* showed the same patterns that higher *D* value should associated with higher *ADC* value in effective group. When b value >200 s/mm^2^, the *ADC* value simply corresponds to *D*, which is the true water diffusion coefficient including both intra- and extracellular water diffusion. Using 4 b values (200, 400, 600, 800 s/mm^2^) to obtain a more accurate *ADC* value, we aimed at exploring whether the difference of ADC values between the two groups was statistical significant or not. But we found that the value of ADC (1.21×10^−3^ mm^2^/s±0.11 vs. 1.33×10^−3^ mm^2^/s±0.23) could not reach statistical significance. While in our study, 5/18 in poor effective group got quirky high value which made the mean *ADC* value illogic high. I even doubted that was the reason why the difference of *ADC* value between the two groups could not reach statistical significance. In future study, when we have enlarged the number of the recruited patients, the result may probably different. Besides, we prefer to obtain a more precise value of *D* and used 800 as the largest b value versus 1000 as the largest b value in other research. I think this might also be another important reason.

In IVIM theory, D* is related to perfusion depended on tumor microvessel attenuation. Furthermore, the value of D* was determined according to the signal intensity ratios of the blood capillaries. In our study, the mean D* value for primary NPC was in agreement with a previous study on metastatic nodes by Lu et al.[16]. D* is large greater than *D*, which explains why D* has a stronger influence on signal decay when b < 200 s/mm2. Our results showed a significant increase in D* (mean, 152.96×10^−3^ mm^2^/s vs. 120.36×10^−3^ mm^2^/s; *P*<0.0001) for effective group than poor effective group. This may result from the differences of histologic structure in the NPC tumor with or without abundant vessels. From the pathological slides of biopsy for the effective group, we found that there was much more neovascularization than the poor effective group. The neovessels could transport the blood and oxygen to the tumor lesions and improve the hypoxia. Obviously, the value of D* in effective groups was higher than poor effective groups, which might result from the more neovascularization in effective groups. Interestingly, the degree of capillary could be revealed by the degree of MRI enhancement in patients with NPC [34]. D* was considered to be proportional to the mean capillary segment length and average blood velocity [9]. So, slow blood velocity and small capillary segment length corresponded to low D* values. While the inadequate blood feed due to the slow blood velocity and small capillary segment length would cause the hypoxia of tumor microenvironment and poor radiosensitivity. Therefore, the increased D* value was proportional to the higher effective radiosensitivity. And D* may be an indicator for evaluating the radiosensitivity of NPC.

Lu, et al claimed that capillary perfusion was increased in malignant tumors [35], while we found *f* (18.79%±2.51 vs. 16.47%±1.51; *P*>0.05) was not significantly different between the two groups. The similar consequence of *f* was previously reported in the fatty liver and hepatocellular carcinoma [25, 36]. One most convinced explanation for this consequence was from Lemke et al. [37], who demonstrated *f* was depended on echo time. The longer echo times caused further signal decays at low b-values, and *f* value increased. This effect would likely be significant for organs with short T2 times. However, in the IVIM sequence, relaxation effects are neglected when these relaxation times diverge, the extracted perfusion-related parameters may depend on TE and TR. Unfortunately, we did not perform T2 calculation, which made us fail to determine the “true” *f* factor.

In order to validate our findings, we had performed the Leave-one-out tests. The classification results obtained from Leave-one out tests of original grouped cases and cross-validated grouped cases were 95.0% and 93.3%. With such high correct classification, IVIM might be a reasonable model to evaluate the radiosensitivity of NPC.

Our study had certain limitations. First, a few cases of NPC, especially for the poor effective group, were recruited in this research. Second, some IVIM images contained some artifacts resulting from physiological motion and inhomogeneous magnetic field at the air-bone and air-soft tissue interfaces in the skull base. Susceptibility artifact was increased with the use of higher b-values, typically approximately 1000 sec/mm^2^. we tried to minimize this effect by setting the maximum b-value as 800 sec/mm^2^. Furthermore, the poor Signal to Noise Ratio (SNR) at higher b-values may decrease the accuracy of IVIM calculation.

In conclusion, *D* and D* were significantly higher in the radiosensitive NPC compared with poor radiosensitive NPC, possibly due to the restriction of molecular diffusion in poor radiosensitive NPC and increased cellularity in radiosensitive NPC. Furthermore, the significantly increased of D* was likely reflecting the increased blood vessel generation and parenchymal perfusion in NPC. These results demonstrated that the biexponential models of IVIM might provide a reasonable model of MRI signal decay in helping evaluate the radiosensitivity of NPC.

## MATERIALS AND METHODS

### Patient selection treatment procedure and assessment

This single-center study was approved by Research Ethics Committee of Guangdong Academy of Medical Sciences. Written informed consent from all patients or their guardians was obtained. From December 2011 to November 2015, 60 consecutive NPC patients (20 female, range, 27-83 years, mean age, 52 years) newly diagnosed by ENT doctor with pharyngeal mirror or nasopharyngo-fiberscope underwent general MRI and IVIM for the primary site to confirm in stage of T3 or T4. All patients accepted a standard regimen of IMRT. The prescribed dose was 69 Gy to planning target volume (PTV) of gross disease in nasopharynx and 67.5 Gy to PTV of positive lymph nodes in 30 fractions, low risk and high risk region PTV was 54 and 60 Gy in 30 fractions, respectively. All the patients were treated with one fraction daily over 5 days/week. Based on the size of locoregional lesion measured on MRI or pharyngorhinoscopy 4 weeks after IMRT, the patients were divided into the effective group and poor effective group. According to the RECIST guideline, complete response (CR) and partial response (PR) were divided into effective group, while no change (NC) and progressive disease (PD) were divided into poor group. In other words, patients with lesion complete nonresidual or lesion decreased more than 50% in area were divided into effective group. And patients with lesion decreased less than 50% or increased were divided into poor group. This study population comprised 42 patients in effective group with tumor remission (CR 35/42, PR 7/42) and 18 patients in poor effective group with tumor enlarged or remain (SD 12/18, PD 6/18).

### Conventional MRI sequence

All the patients were performed with general MRI and IVIM 1-2 days before received IMRT and 4 weeks after the end of IMRT. MRI examinations for nasopharynx were performed with a 3.0-T whole-body system (Signa EXCITE HD, GE Healthcare, Milwaukee, WI, USA) using a 40 mT/m maximum gradient capability and a standard receive-only head and neck coil. The general sequence including axial T_1_WI (TR/TE 600/23 ms; 4 mm thickness, 1 mm gap; NEX=2), axial and coronal contrast-enhanced T_1_WI after a bolus injection of gadolinium diethylenetriamine pentaacetic acid (0.1 mmol/kg, Gd-DTPA; Bayer Healthcare, Berlin, Germany), and axial T_2_WI with fat suppression (TR/TE 5200/137 ms; 4 mm thickness, 1 mm gap; NEX=2) using a 512×288 imaging matrix.

### IVIM imaging sequence

The IVIM sequence was performed before administration of Gd-DTPA. 13 b-values (0, 10, 20, 30, 50, 80, 100, 150, 200, 300, 400, 600 and 800 s/mm^2^) were applied in the sequence with single-shot diffusion-weighted spin-echo echo-planar. The lookup table of gradient directions was modified to allow multiple b-value measurements in one series. Parallel imaging was used with an acceleration factor of 2. A local shim box covering the nasopharynx region was applied to minimize susceptibility artifacts. Totally, 14 axial slices covering the nasopharynx were obtained with a 24 cm field of view, 4 mm slice thickness, 1 mm slice gap, TR/ TE 3000/58 ms, matrix 128×128 and NEX=2. The total scan time was about 225 sec.

### IVIM image analysis

In the bi-exponential model of IVIM sequence, the signal intensities and b-values are related as follows:

$$S_{b}/S_{0}=(1-f)\cdot \;\exp(-\text{b}\cdot D)+f\cdot \exp(-b\cdot D*)$$ (1)

where S_0_ is the signal intensity with b-value of 0; S_b_ is the signal intensity with the b-value denoted by the subscript; *D* is the true diffusion coefficient of a water molecule; D* is the pseudo-diffusion coefficient of microcirculation; and *f* is the micro-vascular volume fraction, indicating the fraction of diffusion related to microcirculation. Based on the assumption that D* is roughly one order of magnitude greater than *D* [38], its influence on signal decay can be neglected for b> 200 s/mm^2^. *f* and D* were calculated by using a non-linear regression algorithm for all b values. At a high b-value (>200 s/mm^2^) -b·D* would be less than −3, and *f*·exp(−b·D*) would be less than 0.05·*f*, and can therefore be neglected. In this case, Eq. (1) can be simplified as follows:

$$S_{b}/S_{0}=(1-f)\cdot \;\exp(-\text{b}\cdot D)$$ (2)

Hence, for high b-values (300, 400, 600, and 800 s/mm^2^) S_b_ was first fitted to Eq. (2) and *D* was calculated. Although we had calculated the *f* value previously, its accuracy was not acceptable; *f* was recalculated using Eq. (1). Then, we fitted S_b_ for all b-values using Eq. (1) with a fixed *D* value using the nonlinear Levenberg-Marquardt method. In the fitting of Eq. (1), the initial estimated values for *f* and D* were set as the previously calculated *f* value from Eq. (2) and 10×10^−3^ mm^2^/s, respectively. Subsequently, *f* and D* were obtained.

ADC value was calculated using a monoexponential fit of (SI) for all the 13 b values:

S(b)=S0exp(−bADC) (3)

### Histogram analysis

Histogram analysis was performed to display the values of all the parameters and their distribution. And such a histogram could be used to analyze the parameters voxel by voxel, thereby providing more precise information than the mean values of the parameters. Following manual lesion segmentation, histograms were generated from each parameter. Voxels for which fits generated unphysical values (<0) were nulled (set to zero) for outlier rejection. Maximum and minimum values were extracted from the distribution.

### Statistical analysis

The mean values of the IVIM parameters were measured independently by two experienced radiologists. They were blinded to the results of IMRT for all patients. First, the axial image section showing the primary tumor at its widest cross-section was determined using T2-weighted images as references. A largest region of interest (ROI) was then manually drawn on axial T2-weighted images by each observer for each tumor at its widest section to cover as much lesions as possible while avoiding the areas of necrosis, air, large vessels, and adjacent anatomical structures (i.e., fat, muscle, and bone), and then subsequently co-registered to IVIM DWI images for further analysis. Each metric value was acquired by each observer and 2 initial data points were generated. The eventual metric value for each tumor was the mean value of the 2 initial data points.

A nonparametric Mann–Whitney test was used to assess IVIM parameters between the effective group and the poor effective group. Receiver Operating Characteristic (ROC) curves was used to estimate the diagnostic tolerance. Youden index (sensitivity-(1- specificity)) was used to found the cutoff point. And we chose the *D* and D* value corresponding to the max value of Youden index as the cutoff point. Intra-class correlation coefficient (ICC) analyse was performed to derive the data variability between the 2 different radiologists. Leave-one-out classification was used to validate our findings. All analyses were performed using SPSS version 13.0 for Windows (SPSS, Chicago, IL, USA). *P*<0.05 was considered statistically significant.

## Abbreviations

IVIM: Intravoxel Incoherent Motion
NPC: Nasopharyngeal Carcinoma
F: =Perfusion Fraction
D*: =Perfusion-related Diffusion
D: Pure Molecular Diffusion
ADC: Apparent Diffusion Coefficient
ROI: Regions of Interest
ICC: Intraclass Correlation Coefficient
IMRT: Intensity-modulated Radiotherapy
DM: Distant Metastasis
DWI: Diffusion-weighted MR Imaging
PTV: Planning Target Volume
ROC: Receiver-operating Characteristic
SNR: Signal to Noise Ratio.
